# Subfamily-selective PCR primers for the human LINE1 L1PA lineage

**DOI:** 10.1038/s41598-025-17649-z

**Published:** 2025-09-12

**Authors:** Marcel Misak, Amitava Basu, Christof Niehrs

**Affiliations:** 1 Institute of Molecular Biology (IMB), 55128 Mainz, Germany; 2 Division of Molecular Embryology, DKFZ-ZMBH Alliance, 69120 Heidelberg, Germany

**Keywords:** L1PA, LINE1, PCR, Primer, Subfamily, Transposition, PCR-based techniques

## Abstract

Long interspersed nuclear element-1 (LINE1) retrotransposons are classified into different subfamilies based on evolutionary history. For human biology, the L1PA lineage of LINE1s is particularly significant. This lineage contains both retrotransposition-competent and inactive subfamilies. PCR-based methods are widely used to monitor LINE1s in genomic DNA. However, PCR analysis distinguishing between L1PA lineage subfamilies of different evolutionary age is thus far challenging due to the difficulty to design primers that are sufficiently specific. Here, we developed and applied a workflow to design PCR primers that discriminate between different subfamilies of the L1PA lineage. Amplicon sequencing after PCR amplification of genomic DNA confirmed that the primers differentiate between groups of L1PA subfamilies of different evolutionary age. We validated the primers using RT-qPCR and verified consistency with publicly available RNA-seq data. Moreover, inhibition of DNA methyltransferase dose-dependently increased LINE1 expression for younger L1PA subfamilies, consistent with transcriptional de-repression. Thus, our primers enable PCR-based surveys that can stratify expression, copy number or epigenetic modification for L1PA subfamilies of differing evolutionary age.

**Supplementary Information:**

The online version contains supplementary material available at 10.1038/s41598-025-17649-z.

## Introduction

LINE1s are the most abundant family of autonomous retrotransposons in the human genome, comprising over 17% of its sequence^1^. These elements play important roles in evolution, embryonic development, and disease^2–4^. In humans, the L1PA lineage of LINE1s is particularly relevant. L1PA contains both retrotransposition-competent and inactive members and has evolved over ~ 100 million years, accumulating mutations that distinguish older L1PA subfamilies from more recently evolved ones^5^. Eighteen L1PA subfamilies have been identified, each representing progressively older waves of L1 retrotransposon activity in primates^5^. These elements have influenced genome diversity, gene regulation, and structural variation, often through insertional mutagenesis or by contributing novel regulatory sequences^6^. Some L1PA subfamilies, particularly L1PA1, often referred to as L1HS, are involved in disease due to their potential for new insertions, leading to mutations and genome instability in conditions such as certain cancers and neurological disorders^7^. L1HS is the most recent active LINE1 subfamily in the human genome and still capable of transposition. Hence, the ongoing expansion of L1HS elements continues to impact human fitness.

Distinguishing between L1PA subfamilies can be critical for understanding how they differ in chromatin and DNA modifications. While profiling different L1PA subfamilies is most accurately performed using sequencing approaches, PCR-based methods would be preferable for high throughput surveys. However, to the best of our knowledge, no experimentally validated PCR primers for distinguishing individual subfamilies within this important lineage have been reported. Indeed, we found that the high sequence homology among L1PA subfamilies, and the substantial sequence diversity even between members of a single subfamily complicate the design of such primers. To overcome these difficulties, we created a workflow to design PCR primers that preferentially amplify sequences of L1PA subfamily members of differing evolutionary age and validated these primers experimentally.

## Results and discussion

**Fig. 1:**
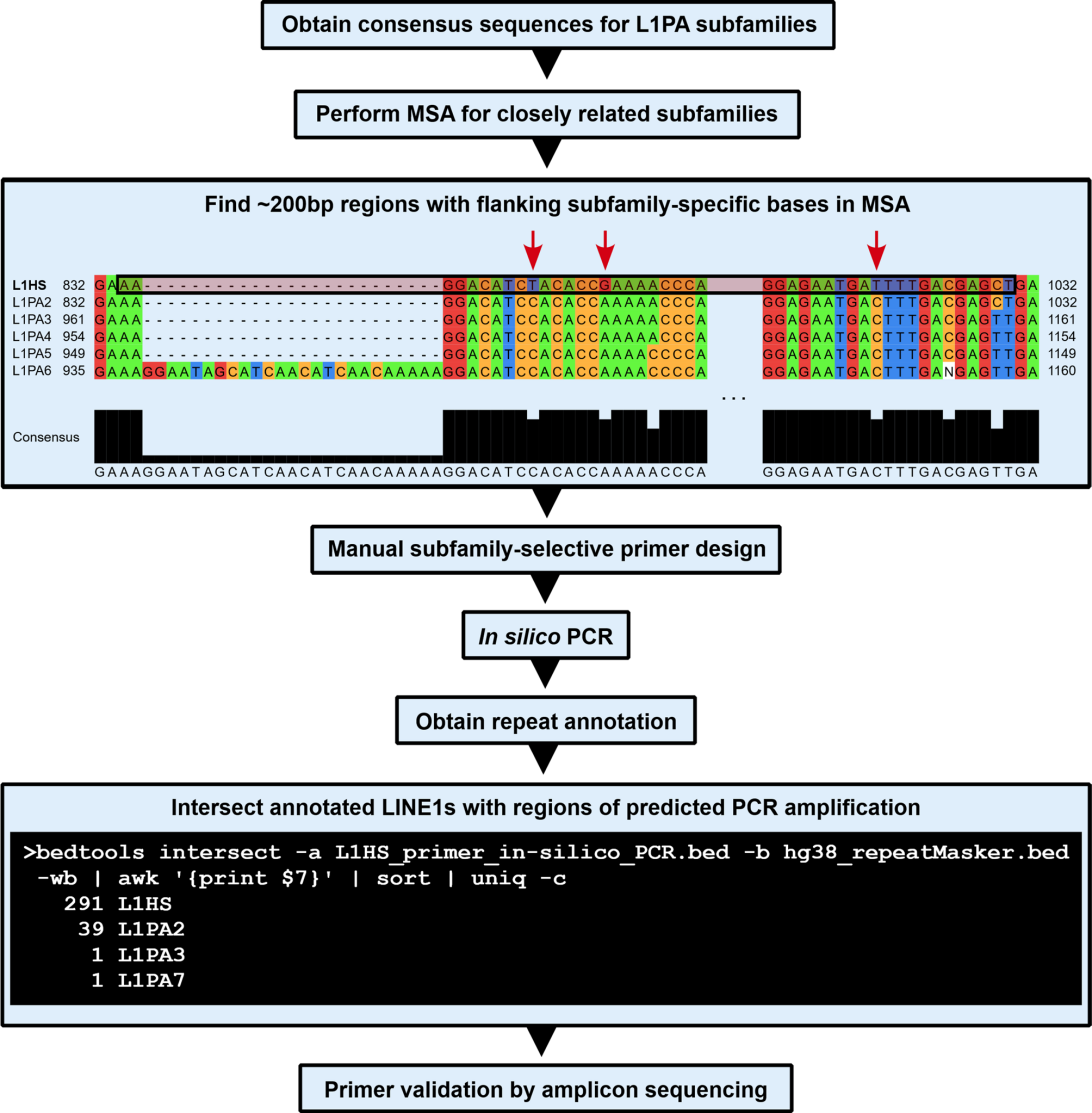
Workflow describing LINE1 subfamily-selective PCR primer design. The primer design process is illustrated using L1PA subfamily L1HS as a representative example.

Figure 1 outlines the design of PCR primers that discriminate between different LINE1 subfamilies in the L1PA lineage, with L1HS shown as a representative example. We obtained consensus sequences for L1HS and evolutionarily close subfamilies L1PA2-L1PA6. We then performed a multiple sequence alignment (MSA) and manually searched for regions of up to ~ 200 bp length that contain L1HS-specific bases at both ends. For regions where primer pairs overlapping L1HS-specific bases could be successfully designed, we predicted PCR targets by *in silico* PCR. We determined that the predicted amplicons substantially overlapped the L1HS annotation, and we subsequently synthesized primer sets. The same methodology was used to design and validate primers for other L1PA subfamilies. In total, we found six primer pairs for which the predicted target regions largely overlapped L1PA subfamilies of interest (Table 1).

**Table 1:** LINE1 subfamily-selective PCR primers. Intended targets are determined based on the selected region in the consensus sequence and the results of *in silico* PCR. Observed targets are based on amplicon sequencing results. Melting temperature according to Eurofins Genomics PCR Primer Design Tool.

Primer name	Intended target	Observed target subfamily(ies)	%GC	Tm	Primer sequence
Primer 1 (F) Primer 1 (R)	L1HS (5’UTR) L1HS (ORF1)	L1HS, L1PA2	50.0 40.9	50.5 50.7	GACATCTACACCGAAAACCC TCGTCAAAATCATTCTCCATCC
Primer 2 (F) Primer 2 (R)	L1PA3 (ORF1) L1PA3 (ORF2)	L1PA2, L1PA3	50.0 50.0	50.2 51.6	ACCAGCCACTGCAAAATC CCAATTTGCCAGTCTGTGTC
Primer 3 (F) Primer 3 (R)	L1PA4 (ORF1) L1PA4 (ORF1)	L1PA4, L1PA5	55.0 52.4	54.6 53.8	ATGCACAAGCCTCAGTAGCC TCCATTCTCCCCGTCACTTTC
Primer 4 (F) Primer 4 (R)	L1PA5 (5’UTR) L1PA5 (ORF1)	L1PA2, L1PA3, L1PA4, L1PA5, L1PA6	52.6 45.5	51.7 51.2	TCCACACCAAAACCCCATC CTCGTCAAAGTCATTCTCCATC
Primer 5 (F) Primer 5 (R)	L1PA16 (ORF2) L1PA16 (ORF2)	L1PA16	40.0 45.0	57.1 58.6	GACAAAGGTGACATTACAAC CTTGGGAGATTGTGTGTTTC
Primer 6 (F) Primer 6 (R)	L1PA17 (ORF2) L1PA17 (ORF2)	L1PA17	45.0 40.0	60.0 56.9	AGAATGAAACTGGACCCCTA GTCCAGAAGAGTATTTCCTA

%GC: Percent GC content, Tm: Melting temperature, F: Forward primer, R: Reverse primer.

**Fig. 2:**
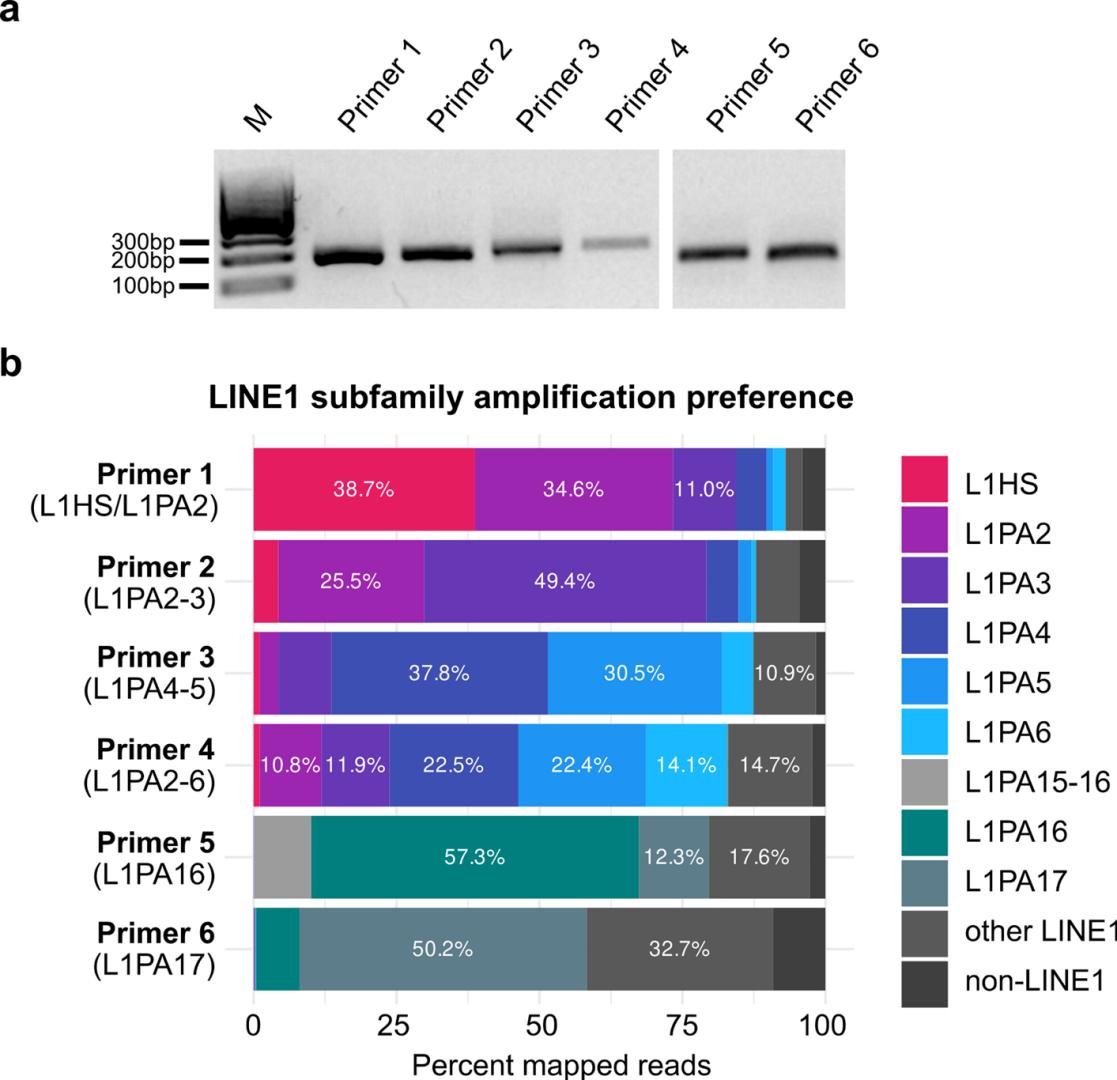
L1PA subfamily-selective PCR primers amplify sequences of interest. (**a**) PCR amplification of human liver genomic DNA using the indicated primers. Agarose gel electrophoresis shows the amplification products. Cropped parts of a single gel are shown, uncropped gel is included in Supplementary Fig. 1. (**b**) Validation of L1PA subfamily-selective primers by amplicon sequencing. Bar chart indicates percentage of all amplified fragments that map to LINE1 and non-LINE1 loci. Predominantly amplified subfamilies are indicated in brackets. Data include multi-mapping reads; a corresponding analysis restricted to unique alignments is presented in Supplementary Fig. 2.

PCR on genomic DNA from human liver yielded distinct 150-200 bp products for all tested primers (Fig. 2a). Amplicon sequencing of these PCR products showed that all primers primarily amplified regions annotated as L1PA sequences (Fig. 2b). While none of the primers was specific to a single L1PA subfamily, primers 1, 2, 3, and 5 predominantly amplified amplicons from two closely related subfamilies by more than ~ 68% and primer 6 amplified a single subfamily by more than 50%. Primer 4, on the other hand, mainly amplified subfamilies L1PA2-L1PA6 with each of these subfamilies contributing between 10.1% and 22.7% of total amplification products (Fig. 2b). These L1PA members emerged after the divergence of Old World primates, but before L1HS, the evolutionarily young and still retrotransposition-competent subfamily^8^. Primer 4 amplicons therefore report members of intermediary evolutionary age. For every primer, a minor share of reads aligned to non-target LINE1 subfamilies or to loci not annotated as LINE1, many of which are likely highly degraded LINE1s and not annotated as such. Supplementary Table 1 shows that both types of off-target loci that are captured by the primers are numerous, yet they account for relatively few reads (Fig. 2b), indicating very shallow amplification per locus. While such off-target amplification could introduce low-level false-positive signal, it is modest relative to the dominant on-target enrichment. When multi-mapping reads are removed (Supplementary Fig. 2), primer 5 shows a modest reduction and primer 6 a pronounced loss of on-target signal, consistent with the fact that these two primers amplify only a small set of highly similar LINE1 loci (Supplementary Table 1) whose products are especially prone to multi-mapping.

**Fig. 3:**
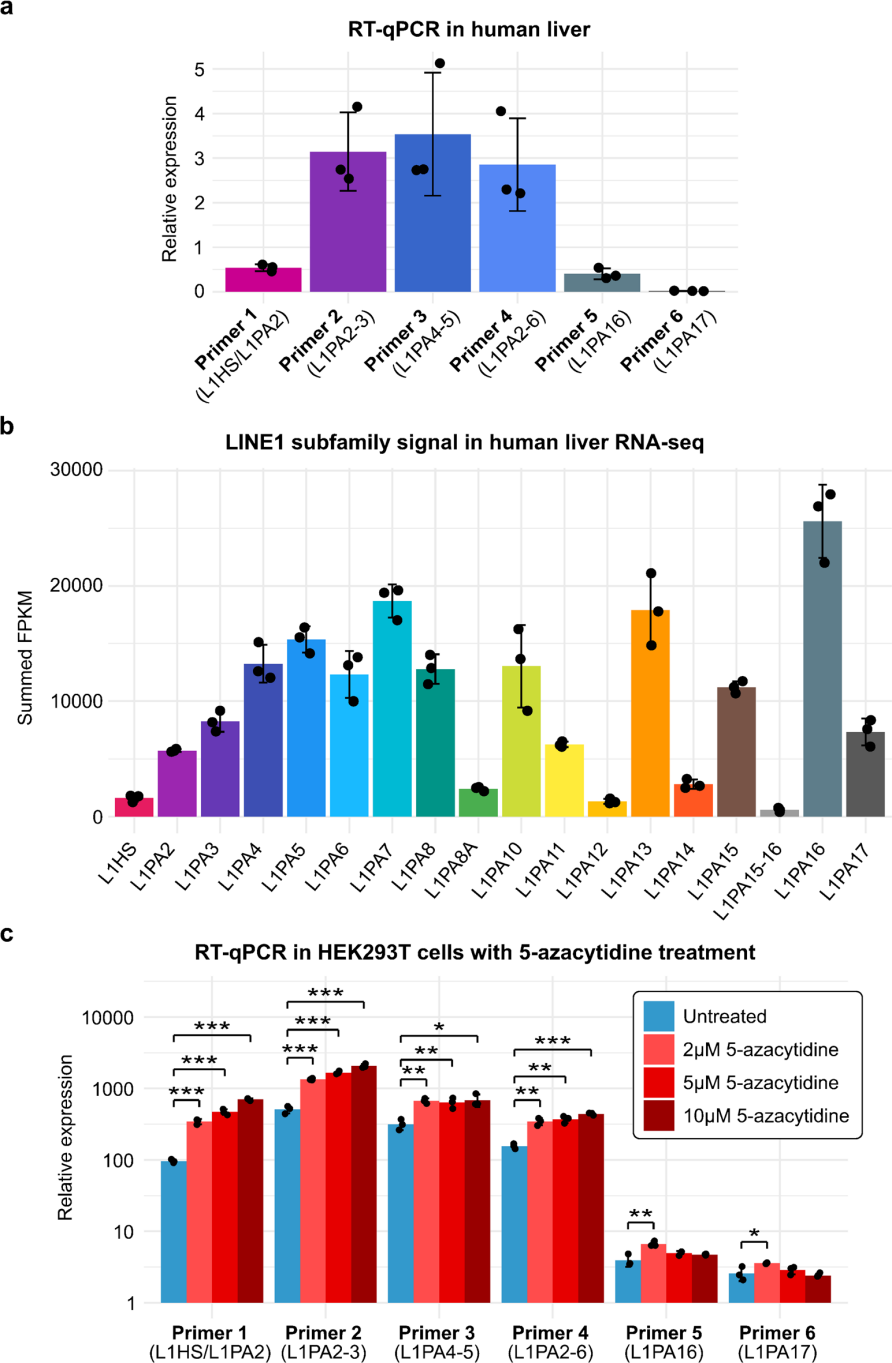
Quantification of L1PA subfamily RNA signals in human liver using subfamily-selective PCR primers. (**a**) LINE1 RT-qPCR analysis of human liver RNA using the indicated primers. Data are presented as mean RT-qPCR levels normalized to *GAPDH* ± standard deviation (s.d.), *n* = 3 technical replicates. (**b**) RNA-seq analysis of L1PA subfamilies in human liver using publicly available data. Summed FPKM values of annotated loci per L1PA subfamily are shown. Data are presented as mean ± s.d., *n* = 3 biological replicates. Data include multi-mapping reads; a corresponding analysis restricted to unique alignments is presented in Supplementary Fig. 3. (**c**) LINE1 RT-qPCR analysis of HEK293T cells following treatment with 5-azacytidine using the indicated primers. Data are presented as mean RT-qPCR levels normalized to *GAPDH* ± standard deviation (s.d.), *n* = 3 biological replicates. Statistical significance was assessed using a two-tailed t-test and *, ** and *** indicate *p* < 0.05, 0.01 and 0.001, respectively.

To corroborate that the primers faithfully amplify their intended targets, we monitored LINE1‑containing transcripts in human liver by RT‑qPCR to compare the results to RNA-seq profiles. Importantly, the primers will indiscriminately detect LINE1 containing RNAs derived from both autonomous and passive transcription. The analysis indicated distinct RT-qPCR signals for different L1PA lineage subfamilies (Fig. 3a). No RT-qPCR signal was observed using primer 6 that overwhelmingly targets L1PA17, the evolutionary oldest L1PA subfamily. Similarly, primer 5 targeting predominantly the second oldest subfamily member, L1PA16, showed only a low signal. Primers 2-4 targeting L1PA2-6 yielded up to 10-fold higher RT-qPCR signal. Primer 1, amplifying L1HS and L1PA2, yielded comparatively weak signals. The observed differences in RT-qPCR signals may be partially explained by subfamily copy numbers: Subfamilies L1PA2-L1PA6 each contain ~ 5,200-12,600 copies, whereas L1HS comprises only ~ 1,700 copies (Supplementary Table 1), suggesting that the per-locus RT-qPCR signal among these subfamilies may be relatively comparable.

We compared the RT-qPCR profiles to RNA-seq data generated from human liver samples^9^ that we reanalyzed for each L1PA subfamily. Consistent with the RT-qPCR results, these data indicate higher RNA-seq signal for L1PA subfamilies L1PA3-6 than for the youngest subfamilies L1HS and L1PA2 (Fig. 3b). However, the oldest L1PA subfamilies L1PA16 and L1PA17 show relatively higher signal than the youngest subfamilies L1HS and L1PA2 in the RNA-seq data compared to RT-qPCR. Since sequence divergence between copies within L1PA subfamilies progressively increases with evolutionary age from L1HS to L1PA17^5^, the primers targeting evolutionarily older subfamilies amplify relatively fewer copies, and thus underestimate their total abundance to a larger extent than primers targeting younger subfamilies (Supplementary Table 1). Furthermore, the reanalyzed RNA-seq datasets included samples from multiple donors with different backgrounds, whereas the RT-qPCR was performed on RNA from a single donor, therefore lacking biological variability. Taken together, crossvalidation of the subfamilyselective primers by RTqPCR with RNAseq datasets confirms their technical robustness.

DNA methylation can silence LINE1s^10,11^ while loss of CpG methylation, e.g. during cellular aging, oncogenic transformation, or upon exposure to DNA-demethylating agents, may reactivate LINE1 promoters and increase their transcription^12–15^. Although our primers cannot resolve autonomous versus passive LINE1 transcription, we nonetheless tested whether these primers can detect transcriptional de-repression of LINE1s. We treated HEK293T cells with different concentrations of the DNA methyltransferase inhibitor 5-azacytidine^16^ and observed a dose-dependent signal increase for primers 1-4 that target evolutionarily younger LINE1 subfamilies, whereas the effects for primers 5 and 6, targeting older subfamilies, were modest in comparison (Fig. 3c).

In addition, these subfamily-selective PCR primers will be suitable for qPCRbased analyses of LINE1 copy number, DNA modifications, or chromatin features. For instance, they can be used to compare LINE1 copy numbers under conditions that de-repress retroelements: primer 1, which captures almost all annotated full-length, intact LINE1s, should report substantial gains, whereas primers 5 and 6 targeting mostly older, defective families could serve as internal controls and are expected to show no change. ChIP-qPCR or DIP-qPCR with the same primer sets can test whether repression of chromatin- or DNA-modifying enzymes produces LINE1 subfamily-specific shifts in chromatin marks or DNA modifications by comparing primer signals across experimental conditions.

## Methods

### PCR primer design

Consensus sequences for L1PA subfamilies L1HS, L1PA2, L1PA3, L1PA4, L1PA5, L1PA6, L1PA14, L1PA15, L1PA16, L1PA17 were obtained from the UCSC Repeat Browser (https://repeatbrowser.ucsc.edu). MSA was performed separately for L1HS, L1PA3, L1PA4, L1PA5, L1PA6 and L1PA14, L1PA15, L1PA16, L1PA17 consensus sequences using Clustal Omega via the EMBL-EBI Job Dispatcher (https://www.ebi.ac.uk/jdispatcher). We manually searched for ~ 200 bp long regions with flanking subfamily-discriminating bases in the MSAs using Jalview v.2.11.4.1. Primers for such regions were designed using Eurofins Genomics PCR Primer Design Tool (https://eurofinsgenomics.eu/en/ecom/tools/pcr-primer-design). PCR was simulated by UCSC’s In-Silico PCR webtool (https://genome.ucsc.edu/cgi-bin/hgPcr) using the designed primers and the GRCh38/hg38 genome assembly. Predicted loci of amplification were converted to BED file format using a custom script and intersected with annotated repeats in the hg38 v.4.0.5 RepeatMasker annotation (https://www.repeatmasker.org/).

### Amplicon sequencing using genomic DNA from human liver

PCR was performed with FastStart™ Taq-DNA-Polymerase (Roche 12032902001) using genomic DNA from human liver cells (Amsbio CD563105). PCR cycle condition: Initial denaturation 95˚C- 10 min, 35 amplification cycles Denaturation 95˚C- 10 s, Annealing 58˚C- 15 s and extension 72˚C- 10 s, and lastly final extension 72˚C- 5 min. PCR products were analyzed on a 2% agarose gel, yielding bands within the expected size range of approximately 200 bp for each primer pair. Bands were excised from the gel and purified using the QIAquick Gel Extraction Kit (Qiagen, Cat. No./ID: 28704). The gel-extracted PCR products were then subjected to amplicon sequencing. DNA library preparation was performed using the NEBNext Ultra II DNA Library Prep Kit from Illumina (Version 6.0, 3/20), with a starting amount of 1.35 ng of DNA. Libraries were amplified through 20 PCR cycles, profiled using a High Sensitivity DNA assay on an Agilent 2100 Bioanalyzer, and quantified with the Qubit dsDNA HS Assay Kit on a Qubit 2.0 Fluorometer (Life Technologies). All samples were pooled in equimolar ratios, purified using AMPure XP Beads (1.0X ratio), and sequenced on a MiSeq Nano Flowcell using paired-end sequencing (2 × 159 cycles), with an additional 7 cycles for the index read. Raw read quality was assessed with FASTQC v.0.11.8 (https://www.bioinformatics.babraham.ac.uk/projects/fastqc) and reads were subsequently mapped to the hg38 human reference genome using Bowtie v.2.3.4 (http://bowtie-bio.sourceforge.net/bowtie2) with options “--very-sensitive --end-to-end --fr --maxins 1000”. To determine the percentages of amplified fragments mapping to LINE1 and non-LINE1 loci for each primer, read counting was performed using Subread featureCounts v.2.0.0 (https://subread.sourceforge.net). All properly paired fragments that overlapped with either a LINE1 subfamily in the hg38 v.4.0.5 RepeatMasker annotation (https://www.repeatmasker.org/) or with non-LINE1 genomic loci were counted. To estimate the number of genomic loci captured by each primer, we first generated four annotation tracks: (i) all LINE1s in the hg38 RepeatMasker v4.0.5, (ii) the genomic complement of these LINE1 coordinates (non-LINE1), (iii) full-length LINE1s (> 6 kb in length), and (iv) the subset of those full-length LINE1s that intersect copies classified as full-length and intact according to L1Base (https://l1base.charite.de), using the annotation for NCBI38. Read pairs were filtered with SAMtools v1.17 (http://www.htslib.org) to retain only uniquely aligned fragments, and a BED file was compiled from intervals supported by at least three fragments in either the filtered or unfiltered alignments. These intervals were designated “amplified loci” and intersected with each of the four previously generated annotation tracks.

### Human liver RT-qPCR

cDNA synthesis was performed on human liver RNA (Amsbio R1234151-50) with SuperScript II Reverse Transcriptase (ThermoFisher). RT-qPCR was performed on a LightCycler 480 (Roche) in technical triplicate RNA samples with each reaction performed in duplicates using LightCycler 480 SYBR Green I Master. Quantitative analysis was performed with LightCycler 480 software v.1.5.1.62 (https://lifescience.roche.com/global/en/products/product-category/lightcycler.html). PCR was performed using the indicated L1PA subfamily-selective primers. For *GAPDH*, the following primer pair was used: Forward 5′ACCCAGAAGACTGTGGATGG-3′; Reverse, 5′TTCAGCTCAGGGATGACCTT-3′. A no-reverse transcriptase control was included for all RT-qPCR reactions to confirm the absence of amplification from residual genomic DNA; these control samples consistently showed higher cycle threshold values (i.e. lower signals) compared to the reactions containing reverse transcriptase.

### HEK293T RT-qPCR with 5-azacytidine treatment

HEK293T (ATCC, CRL-11268) were cultured in DMEM (Gibco) supplemented with 10% FBS Gold (PAA), 2 mM L-glutamine, and 100 U/ml PEN-STREP at 37 °C in 5% CO2 and 20% O2. Treatment of the HEK293T cells was performed with 2µM, 5µM and 10µM of 5-azacytidine. The cells were treated for 5 days and media with and 5-azacytidine was changed every day. HEK293T cells were grown in triplicates for each condition, including a control group with no treatment. RNA was isolated after 5 days followed by cDNA synthesis and qPCR was performed as described above for all samples using the indicated primers.

### Human liver RNA-seq analysis

RNA-seq data generated from healthy human liver was obtained from ENA (ERR3668588, ERR3668589 and ERR3668590). Raw read quality was assessed with FASTQC v.0.11.8 (https://www.bioinformatics.babraham.ac.uk/projects/fastqc) and reads were subsequently mapped to the hg38 human reference genome using STAR v.2.7.3a (https://github.com/alexdobin/STAR). Read counting was performed using Subread featureCounts v.2.0.0 (https://subread.sourceforge.net). All properly paired, strand-specific fragments whose orientation matched that of a LINE1 subfamily in the hg38 RepeatMasker v.4.0.5 (https://www.repeatmasker.org/) were counted. RPKM values were computed for each LINE1 locus and summed up per LINE1 subfamily for each RNA-seq sample.

## Supplementary Information

Below is the link to the electronic supplementary material.

Supplementary Material 1

## Data Availability

Amplicon sequencing data for each primer is available on SRA under accession number PRJNA1241216 (https://www.ncbi.nlm.nih.gov/bioproject/PRJNA1241216).
