# Supplementary material for: Subfamily-selective PCR primers for the human LINE1 L1PA lineage
Source: Sci Rep. 2025 Sep 12;15:32499. doi: 10.1038/s41598-025-17649-z (PMC12432230; doi:10.1038/s41598-025-17649-z)
Supplement: Supplementary file 1 — Supplementary Material 1 [file 41598_2025_17649_MOESM1_ESM.pdf]

Supplementary information

Subfamily-Selective PCR Primers for the Human LINE1 L1PA Lineage

Marcel Misak[1], Amitava Basu[1] and Christof Niehrs[1,2,3]

[1]Institute of Molecular Biology (IMB), 55128 Mainz, Germany

[2]Division of Molecular Embryology, DKFZ-ZMBH Alliance, 69120 Heidelberg, Germany

[3]Corresponding author: c.niehrs@imb-mainz.de

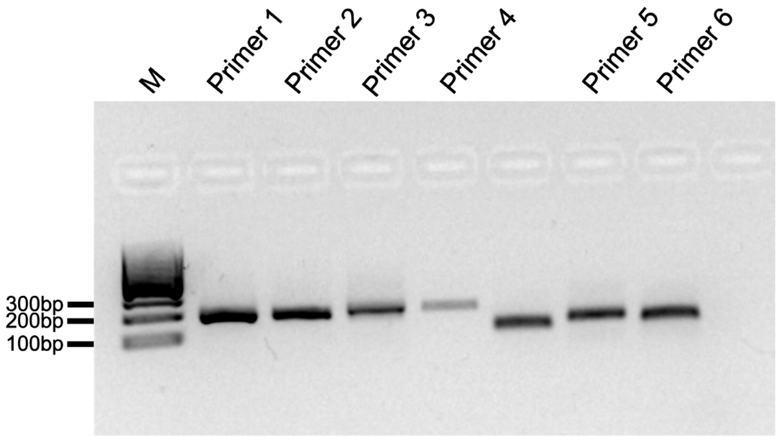

Supplementary Figure 1: PCR amplification of human liver genomic DNA using the indicated primers. Agarose gel electrophoresis shows the amplification products. Uncropped agarose gel showing all lanes and molecular weight markers, corresponding to the cropped gel parts presented in Figure 2a.

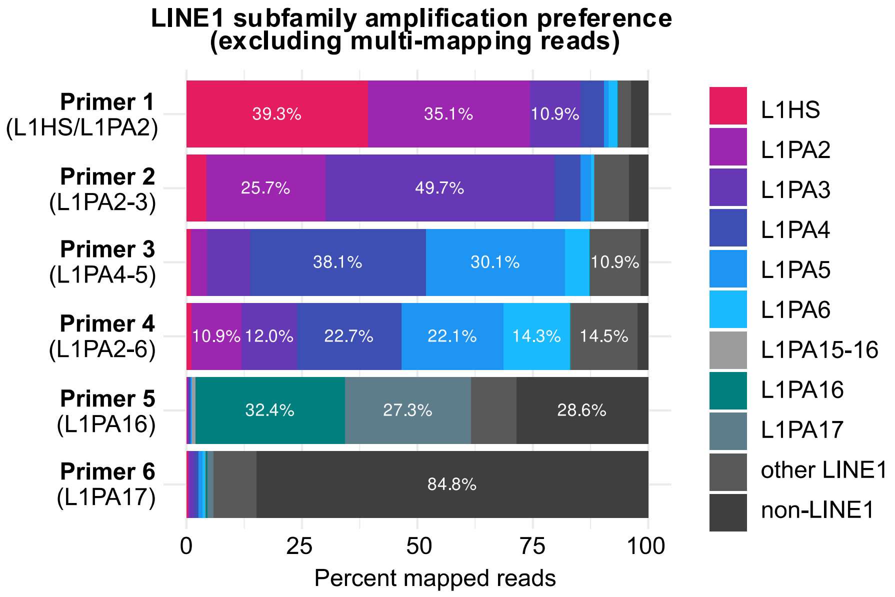

Supplementary Figure 2: Amplicon-sequencing validation of L1PA-selective primers after removal of multimapping reads. Validation of L1PA subfamily-selective primers by amplicon sequencing. Bar chart indicates percentage of all amplified fragments that map to LINE1 and non-LINE1 loci. Serving as a direct comparison to Figure 2b, this plot reflects data after the exclusion of multi-mapping reads.

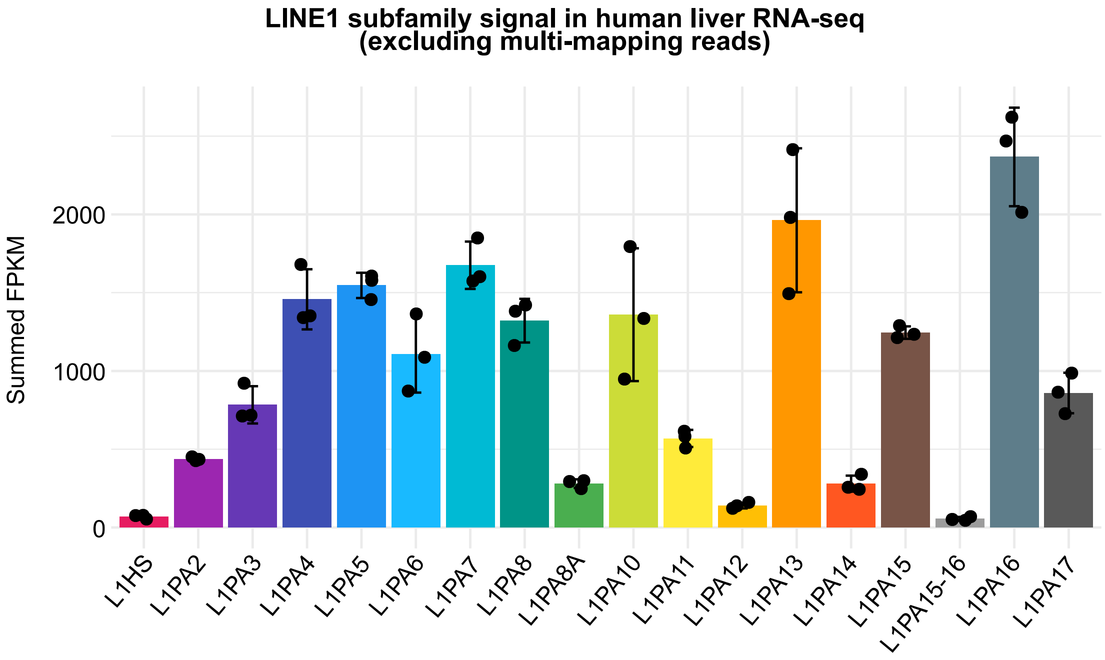

Supplementary Figure 3: RNA-seq analysis of L1PA subfamilies after removal of multi-mapping reads. Summed FPKM values of annotated loci per L1PA subfamily are shown. Data are presented as mean ± s.d., n = 3 biological replicates. Serving as a direct comparison to Figure 3b, this plot reflects data after the exclusion of multi-mapping reads.

Supplementary Table 1: Number of loci amplified per LINE subfamily for each LINE1 subfamily-selective PCR primer. Counts derived from amplicon sequencing of genomic DNA extracted from human liver are presented per subfamily and further categorized by locus type: all, full-length (FL; >6kb), and FL with intact ORF regions (FL & intact). Non-LINE1 loci represent the complement of annotated LINE1 elements in the human genome. Ranges reflect counts without and with multi-mapping reads, respectively; identical values are shown as single entries.

Subfamily	Type	Total	Primer 1 (L1HS/L1PA2)	Primer 2 (L1PA2-3)	Primer 3 (L1PA4-5)	Primer 4 (L1PA2-6)	Primer 5 (L1PA16)	Primer 6 (L1PA17)
**L1HS**	All	1713	380-383	168-175	150-162	75-78	0	0
	FL	327	302-305	43-45	69-75	15-18	0	0
	FL & intact	104	102	12-13	17-20	1-2	0	0
**L1PA2**	All	5215	773-785	852-868	583-608	695-722	0	1
	FL	1015	660-671	712-723	490-511	594-617	0	1
	FL & intact	33	26-27	20-22	12	12-15	0	0
**L1PA3**	All	11298	658-676	1449-1462	1079-1122	954-993	0-1	0
	FL	1425	526-541	1047-1055	829-862	759-789	0	0
	FL & intact	0	0	0	0	0	0	0
**L1PA4**	All	12558	386-407	337-353	1160-1193	785-797	1-2	1-2
	FL	1305	283-300	239-251	867-886	586-595	1	0-1
	FL & intact	0	0	0	0	0	0	0
**L1PA5**	All	11809	170-189	180-188	833-858	623-636	1	1
	FL	900	113-126	103-107	545-558	407-416	0	0
	FL & intact	0	0	0	0	0	0	0
**L1PA6**	All	6230	164-178	102-107	249-263	431-436	0	1
	FL	744	97-105	52-55	155-165	264-268	0	0
	FL & intact	0	0	0	0	0	0	0
**L1PA15-16**	All	1482	0	1	0	0	4-19	0
	FL	4	0	0	0	0	0	0
	FL & intact	0	0	0	0	0	0	0
**L1PA16**	All	14714	3	4	1	2	34-136	1-25
	FL	42	0	0	0	0	0	0-1
	FL & intact	0	0	0	0	0	0	0
**L1PA17**	All	4937	3	2	0	1	4-29	2-40
	FL	11	0	0	0	0	0	0
	FL & intact	0	0	0	0	0	0	0
**Other LINE1**	All	952133	230	588	415	484	13	6
	FL	1928	18	98	17	64	0	0
	FL & intact	0	0	0	0	0	0	0
**Non-LINE1**	All	915988	266	572	80	36	54	50
